# The role of plasminogen activator inhibitor-1 in gastric mucosal protection

**DOI:** 10.1152/ajpgi.00017.2013

**Published:** 2013-03-14

**Authors:** Susan Kenny, Islay Steele, Suzanne Lyons, Andrew R. Moore, Senthil V. Murugesan, Laszlo Tiszlavicz, Rod Dimaline, D. Mark Pritchard, Andrea Varro, Graham J. Dockray

**Affiliations:** ^1^Institute of Translational Medicine, University of Liverpool, Liverpool, United Kingdom; and; ^2^Department of Pathology, University of Szeged, Szeged, Hungary

**Keywords:** stomach, nonsteroidal anti-inflammatory drugs, aspirin, gastric lesion, hemostasis

## Abstract

Gastric mucosal health is maintained in response to potentially damaging luminal factors. Aspirin and nonsteroidal anti-inflammatory drugs (NSAIDs) disrupt protective mechanisms leading to bleeding and ulceration. The plasminogen activator system has been implicated in fibrinolysis following gastric ulceration, and an inhibitor of this system, plasminogen activator inhibitor (PAI)-1, is expressed in gastric epithelial cells. In *Helicobacter pylori*-negative patients with normal gastric histology taking aspirin or NSAIDs, we found elevated gastric PAI-1 mRNA abundance compared with controls; the increase in patients on aspirin was independent of whether they were also taking proton pump inhibitors. In the same patients, aspirin tended to lower urokinase plasminogen activator mRNA. Immunohistochemistry indicated PAI-1 localization to epithelial cells. In a model system using MKN45 or AGS-G_R_ cells transfected with a PAI-1 promoter-luciferase reporter construct, we found no evidence for upregulation of PAI-1 expression by indomethacin, and, in fact, cyclooxygenase products such as PGE_2_ and PGI_2_ weakly stimulated expression. Increased gastric PAI-1 mRNA was also found in mice following gavage with ethanol or indomethacin, but plasma PAI-1 was unaffected. In PAI-1^−/−^ mice, gastric hemorrhagic lesions in response to ethanol or indomethacin were increased compared with C57BL/6 mice. In contrast, in PAI-1-H/Kβ mice in which PAI-1 is overexpressed in parietal cells, there were decreased lesions in response to ethanol and indomethacin. Thus, PAI-1 expression is increased in gastric epithelial cells in response to mucosal irritants such as aspirin and NSAIDs probably via an indirect mechanism, and PAI-1 acts as a local autoregulator to minimize mucosal damage.

gastric mucosal health is maintained in the presence of a hostile luminal environment consisting of acid, potentially damaging chemical and physical factors in the diet, and resident and ingested microbiota. Mucosal protection depends on multiple mechanisms operating at both epithelial and subepithelial levels; these include mucus and bicarbonate secretion by epithelial cells, migration (restitution) and renewal of epithelial cells, epithelial apical membrane properties, maintenance of junctional assemblies between epithelial cells, regulation of mucosal blood flow at least partly via neuronal axon reflexes, and wound healing responses by stromal cells such as myofibroblasts, inflammatory cells, and immune cells (13). The collective importance of these mechanisms is well illustrated by the adverse responses to aspirin or nonsteroidal anti-inflammatory drugs (NSAIDs). These inhibit cyclooxygenase (COX)-1 and -2 expressed both in stromal and epithelial cells, the products (prostaglandins, PGI_2_, and thromboxane A_2_) of which regulate epithelial bicarbonate secretion, blood flow, and inflammatory responses (24). Gastric bleeding in patients treated with aspirin or NSAIDs remains a major clinical problem that may well have overtaken *Helicobacter pylori* as a cause of peptic ulcer disease in some settings (16).

Tissue responses to damage involve distinct phases of hemostasis, inflammation, proliferation, and tissue remodeling (19). The disruption of these mechanisms that occurs with aspirin and NSAIDs is complex and multifactorial. There is evidence, for example, for both prostaglandin-dependent and -independent components in the responses to NSAIDs (16), as well as the involvement of other signaling molecules (chemokines, cytokines, and growth factors) and interactions with systems regulating tissue architecture, including deposition of extracellular matrix proteins and their degradation by matrix metalloproteinases and other extracellular proteolytic systems. The latter, in particular, include the tissue and urokinase plasminogen activators (tPA and uPA, respectively) that convert plasminogen to plasmin, which in turn degrades fibrin. The early activation of tPA and uPA inhibits hemostasis by fibrin degradation while their later activation contributes to tissue remodeling. Plasminogen activator activity is restrained by several inhibitors, the most important of which is plasminogen activator inhibitor (PAI)-1 (5).

Recent studies have identified expression of PAI-1 in gastric epithelial cells and have shown increased expression with *Helicobacter pylori* infection (9, 10) and with elevated plasma gastrin (18). Expression of PAI-1 has been linked to inhibition of gastric epithelial cell proliferation via suppression of the release of heparin-binding epithelial growth factor by uPA, and to inhibition of epithelial cell migration (10, 18). However, the biology of PAI-1 is complex, and there is also evidence for actions that are independent of tPA or uPA (15). In the present study, we hypothesised that, given the regulated expression of PAI-1 in the gastric mucosa and its potential effects on several different aspects of wound healing, it would play a role in gastric mucosal protection. In view of the effect of aspirin and NSAIDs in disrupting gastric mucosal protection, we first examined PAI-1 mRNA abundance in patients receiving aspirin or NSAIDs and then the role of PAI-1 in two experimental models of mucosal protection. We report here elevated PAI-1 mRNA in patients taking aspirin or NSAIDs and a protective effect of PAI-1 in two experimental models of gastric mucosal damage.

## MATERIALS AND METHODS

### 

#### Cells, plasmids, and reagents.

AGS-G_R_ cells were maintained as previously described (22). MKN45 gastric carcinoma cells were purchased from the American Type Culture Collection and cultured in Dulbecco's Modified Eagle Medium (DMEM) supplemented with 10% fetal bovine serum (FBS), 1% penicillin-streptomycin, and 2% antibiotic-antimycotic solution. Human gastric corpus myofibroblasts that had been prepared from normal gastric mucosa of transplant donors as previously reported (6) were maintained in DMEM supplemented with 10% FBS, 1% nonessential amino acid solution, 1% penicillin-streptomycin, and 2% antibiotic-antimycotic. In coincubation experiments, AGS-G_R_ cells had been adapted through several passages to grow in DMEM. A plasmid encoding 4.5 kb of the human PAI-1 promoter coupled to luciferase (PAI-1-luc) has previously been reported (10). A monoclonal antibody to PAI-1 was obtained from American Diagnostica (Stamford, CA). A constitutively active *Renilla* luciferase plasmid (pRL-Sv40), TransFast transfection reagent, and a dual luciferase assay kit were purchased from Promega (Southampton, Hants, UK). Gastrin-17 (G-17) was obtained from Bachem (St. Helens, Merseyside, UK). Phorbol 12-myristate, 13-acetate (PMA), absolute ethanol, and indomethacin (dissolved in 5% sodium bicarbonate) were obtained from Sigma (Poole, Dorset, UK). ^125^I-labeled G-17 was purchased from PerkinElmer (Cambridge, Cambs, UK).

#### Patients.

Subjects were selected from a cohort of ∼1,400 patients who were age 18 and above and had clinical indications for undergoing upper gastrointestinal endoscopy. General exclusion criteria for the cohort as a whole included coma or hemodynamic instability, being moribund, or having terminal malignancy, cirrhosis (Child B or C), abnormal clotting or bleeding diasthesis, inability to give informed consent, contraindication to endoscopy, pregnancy, human immunodeficiency virus, or hepatitis B or C infections. Subjects were then selected for the current investigation if they were *H. pylori* negative (see below) and additionally showed no endoscopic or histological evidence of upper gastrointestinal neoplasia, preneoplastic pathology (atrophic gastritis, gastric intestinal metaplasia, or Barrett's esophagus), or diabetes mellitus. Subjects underwent diagnostic gastroscopy in the gastroenterology unit at the Royal Liverpool and Broadgreen University Hospitals NHS Trust. Endoscopic pinch biopsies of gastric corpus and antrum (2–4 of each) were obtained for histology, and *H. pylori* status was determined on the basis of serology, antral urease test (Pronto Dry; Medical Instrument Corporation, Solothurn, Switzerland), and antral and corpus histology. An additional eight corpus biopsies were taken for RNA extraction and real-time PCR analysis. The study groups consisted of *H. pylori*-negative patients with normal gastric histology taking either aspirin (43, all on 75 mg daily) or NSAIDs [8, ibuprofen (200–600 mg); 5, diclofenac (150 mg); 2, naproxen; 1, meloxicam; 1, celecoxib; 1, arthrotec (diclofenac and misoprostol)]. In the aspirin group, 19 were taking proton pump inhibitors [PPIs: 6, omeprazole (20–40 mg daily); 13, lansoprazole (15–30 mg daily)]; in the NSAID group 12 were on PPIs [5, omeprazole (20 mg); 5, lansoprazole (30 mg); 1, esomeprazole (20 mg); 1 pantoprazole (20 mg)]. Age- and sex-matched controls were *H. pylori* negative and had normal gastric histology; they included patients both off and on PPIs [10, omeprazole (20–40 mg); 15, lansoprazole (15–30 mg); 1, pantoprazole (20 mg); 1, rabeprazole (20 mg)]. The study was approved by the Liverpool Local Research Ethics Committee and by the Royal Liverpool and Broadgreen University Hospitals NHS Trust, and all patients gave written, informed consent.

#### Animals.

Wild-type C57BL/6 mice were obtained from Charles River (Margate, Kent, UK). Mice null for PAI-1 (PAI-1^−/−^) on a C57BL/6 background were purchased from JAX MICE. Mice with targeted expression of PAI-1 to the stomach (PAI-1-H/Kβ mice) were produced as previously described (11). All mice were individually housed, and an equal mix of males and females was used at 10–12 wk old. Animals were maintained in an appropriately controlled environment with a 12:12-h light-dark cycle and were fed a commercial pellet diet with water ad libitum. Animals were killed by increasing CO_2_ concentration. All animal experiments were approved by the University of Liverpool Animal Welfare Committee and were conducted in compliance with Home Office requirements and the United Kingdom Animals (Scientific Procedures) Act 1986.

#### Real-time PCR.

Human corpus pinch biopsies and whole-thickness mouse corpus tissue were collected in RNA Later (Life Technologies, Paisley, UK), and RNA was extracted in 1.0 ml Tri-Reagent (Sigma) according to the manufacturer's instructions; RNA pellets were resuspended in 30 μl of nuclease-free water, and 2 μg of RNA were reverse transcribed with avian myeloblastosis virus reverse transcriptase and oligo(dT) primers (Promega). Real-time PCR was carried out using an ABI7500 platform (Applied Biosystems, Warrington, UK) using TaqMan primer/probe sets (human PAI-1, PAI-2, uPA, and GAPDH), Precision 2× real-time PCR master mix (Primer Design, Southampton, UK), and 5′-FAM, 3′-TAMRA double-dye probes (Eurogentec, Southampton, UK) or using SYBR green (mouse PAI-1 and GAPDH). Quantifications were performed using a standard curve, and all values were standardized to GAPDH determined in the same sample. Assays included a no template control and three quality controls and were only accepted if they met the following criteria: the quality controls fell within 15% of their anticipated mean quantity, the PCR amplification efficiency was between 90 and 110%, and the correlation coefficient of the slope of the standard curve was >0.97. Primers and probes were designed using Primer Express version 3.0 (Applied Biosystems) and were purchased from Eurogentec (Seraing, Belgium). Probes for detection of human PAI-1, PAI-2, uPA, and GAPDH mRNA were intron-spanning as were primers for detection of mouse PAI-1 and GAPDH mRNA. Primer and probe sequences were as follows: human PAI-1: 5′-TGC CCA TGA TGG CTC AGA-3′ (forward), 5′-GCA GTT CCA GGA TGT CGT AGT AAT G-3′ (reverse), 5′-AGT TCA ACT ATA CTG AGT TCA CCA CGC CCG-3′ (probe); human PAI-2: 5′-GGC CAA GGT GCT TCA GTT TAA T-3′ (forward), 5′-TGA ACC CAC AGC TGG TAA AGT TC-3′ (reverse), 5′-CCA ATG CAG TTA CCC CCA TGA CTC CA-3′ (probe); human uPA: 5′-ACA CTG CTT CAT TGA TTA CCC AAA-3′ (forward), 5′-CCC CTT GCG TGT TGG AGT T-3′ (reverse), 5′-ATC GTC TAC CTG GGT CGC TCA AGG CT-3′ (probe); human GAPDH: 5′-GCT CCT CCT GTT CGA CAG TCA-3′ (forward), 5′-ACC TTC CCC ATG GTG TCT GA-3′ (reverse), 5′-CGT CGC CAG CCG AGC CAC A-3′ (probe); mouse PAI-1: 5′-CTG CAG ATG ACC ACA GCG GG-3′ (forward), 5′-AGC TGG CGG AGG GCA TGA-3′ (reverse); and mouse GAPDH: 5′-TCT TGT GCA GTG CCA GCC TC-3′ (forward), 5′-TGG CAG CCC TGG TGA CCA-3′ (reverse).

#### Gastrin radioimmunoassay.

Human serum samples were assayed for total amidated gastrin concentrations by radioimmunoassay using antibody L2 (which reacts with G-17 and G-34 but not progastrin or Gly gastrins) and ^125^I-labeled G-17 as previously described (4).

#### Immunohistochemistry.

Tissue sections from gastric corpus biopsies fixed in 10% neutral-buffered formalin and paraffin-embedded were processed for immunohistochemical detection of PAI-1 after antigen recovery as previously described (21).

#### Luciferase promoter-reporter assays.

AGS-G_R_ or MKN45 cells (2 × 10^5^) were plated in six-well plates in full medium for 24 h before being transfected with PAI-1-luc (1 μg/well) and a constitutively active *Renilla* luciferase plasmid as an internal control (pRL-Sv40, 0.5 ng/well) using TransFast Transfection Reagent according to the manufacturer's instructions. After transfection, cells were incubated in full media for 24 h before being stimulated in serum-free media with 100 ng/ml PMA, 10 nM human unsulfated G-17, 100 mM indomethacin, or other compounds for 6 h as indicated. In addition, 24 h after transfection, AGS-G_R_ cells were coincubated with gastric myofibroblasts (5 × 10^4^) for 24 h before being stimulated in serum-free media with 10 nM G-17 or 100 mM indomethacin for 6 h. Luciferase activity was measured by dual-luciferase assay according to the manufacturer's instructions in a Lumat LB9507 luminometer (Berthold, Herts, UK). Results are presented as fold increase over unstimulated control, so the value of 1.0 signifies no change in luciferase activity.

#### Gastric hemorrhagic lesion models.

Male or female C57BL/6, PAI-1^−/−^, and PAI-1-H/Kβ mice were fasted overnight (17 h) before being gavaged with saline (vehicle), absolute or 50% ethanol, or with 5% sodium bicarbonate (vehicle) or 20 mg/kg indomethacin (100 μl). Animals were killed 1 h after ethanol or 6 h after indomethacin treatment. Stomachs were removed, opened along the lesser curvature, gently washed in phosphate-buffered saline, and fully stretched out mucosa side up. The number and length of hemorrhagic lesions was recorded using callipers, and the total lesion score (mm) per animal was calculated. Stomachs were photographed using a Canon Ixus izoom camera (Canon, Surrey, UK). Gastric corpus tissue from vehicle and ethanol- or indomethacin-treated C57BL/6 mice was collected for RNA extraction, and real-time PCR was carried out as described above.

#### PAI-1 enzyme-linked immunosorbent assay.

Blood was collected in 0.1 M trisodium citrate via cardiac puncture from C57BL/6 mice 1 h postgavage with saline or absolute ethanol and 6 h post vehicle or indomethacin treatment. Plasma concentrations of PAI-1 were determined by enzyme-linked immunosorbent assay (ELISA; Molecular Innovations) according to the manufacturer's instructions.

#### Statistics.

Results are presented as means ± SE; comparisons were made using ANOVA or Student's *t*-test where appropriate and were considered significant at *P* < 0.05.

## RESULTS

### 

#### Aspirin or NSAIDs increase gastric PAI-1 mRNA.

**Fig. 1.**
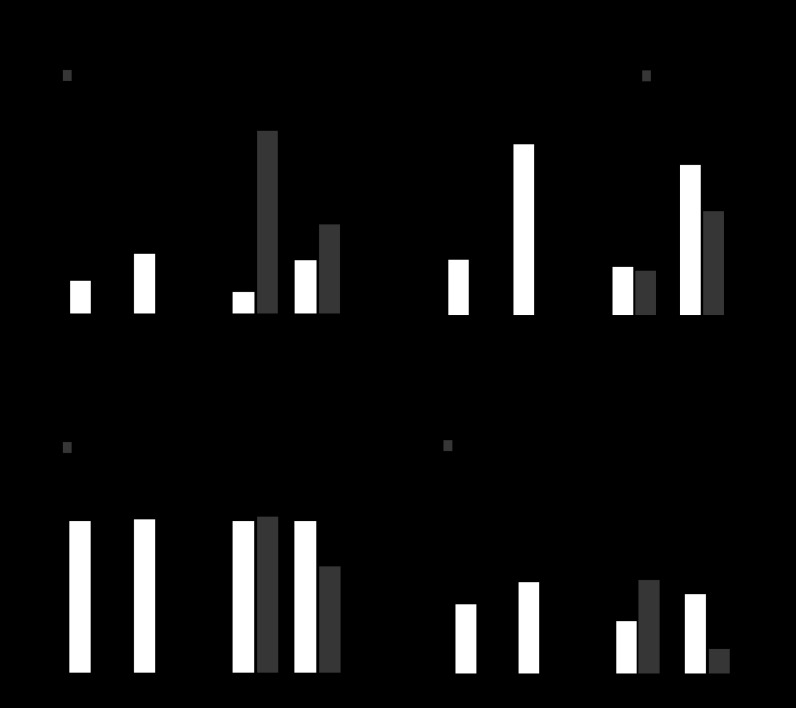
Increased plasminogen activator inhibitor (PAI)-1, but not urokinase plasminogen activator (uPA) or PAI-2, mRNA in the gastric corpus of subjects using aspirin or nonsteroidal anti-inflammatory drugs (NSAIDs). *A*: increased abundance of PAI-1 mRNA relative to GAPDH mRNA in gastric biopsies from patients receiving either aspirin [mean age: 68.5 ± 1.6, body mass index (BMI): 26.6 ± 0.7; controls: 68.0 ± 1.6, BMI: 28.2 ± 0.9; 23 females, 20 males] or NSAIDs (mean age: 58.0 ± 2.0, BMI: 29.5 ± 1.0; controls: 57.3 ± 1.7, BMI: 26.7 ± 0.9; 10 females, 7 males) with or without proton pump inhibitors (PPIs). *B*: serum gastrin concentration is not increased by aspirin or NSAIDs but is significantly increased by PPI use. *C*: gastric uPA mRNA abundance relative to GAPDH is reduced in patients receiving aspirin regardless of PPIs and unchanged in patients on NSAIDs. *D*: gastric PAI-2 mRNA abundance relative to GAPDH is not significantly different in patients receiving aspirin or NSAIDs, regardless of PPI usage, compared with the relevant controls. *P < 0.05, ANOVA.

In initial studies, we examined gastric PAI-1 mRNA abundance in a cohort of patients receiving either aspirin or NSAIDs. There was a three- to fivefold higher abundance of PAI-1 mRNA relative to GAPDH mRNA in both the aspirin (0.056 ± 0.009 vs. 0.199 ± 0.020; mean ± SE, *n* = 43, *P* < 0.001) and NSAID (0.038 ± 0.008 vs. 0.191 ± 0.050, *n* = 17, *P* < 0.001) groups compared with age- and sex-matched controls. Many patients were also receiving PPIs, and, since PPIs increase plasma gastrin which may also stimulate PAI-1 expression (18), we then refined the analysis to take this into account (Fig. 1*A*). Patients taking aspirin together with PPIs had significantly higher serum gastrin concentrations compared with those not on PPIs (Fig. 1*B*) and so too did control patients; in the NSAID group, however, the difference in serum gastrin concentrations between those on and off PPIs was not significantly different. In patients on aspirin, the relative abundance of PAI-1 mRNA in those taking PPIs was not significantly different from that in those not taking PPIs (Fig. 1*A*). In patients on NSAIDs, the relative increase in PAI-1 mRNA compared with the appropriate control group was only seen in those who were not on PPIs; in those on PPIs the difference was not statistically significant (Fig. 1*A*). In control subjects, PPI use was associated with a twofold increase in PAI-1 mRNA, but this was not statistically significant. The data suggest that aspirin usage is associated with a robust increase in gastric mucosal PAI-1 abundance independent of PPI usage or serum gastrin concentrations; for patients on NSAIDs, but not PPIs, PAI-1 mRNA is also increased.

To determine the specificity of the PAI-1 mRNA response, we then determined uPA and PAI-2 mRNA abundances. There was a significant decrease in uPA mRNA in patients taking aspirin either with or without PPIs, although there was no difference in patients on NSAIDs (Fig. 1*C*). In the case of PAI-2, there were no significant differences in relative abundance in patients on aspirin or NSAIDs, with or without PPIs, compared with the appropriate controls (Fig. 1*D*).

#### Gastric epithelial localization of PAI-1 with aspirin or NSAID treatment.

**Fig. 2.**
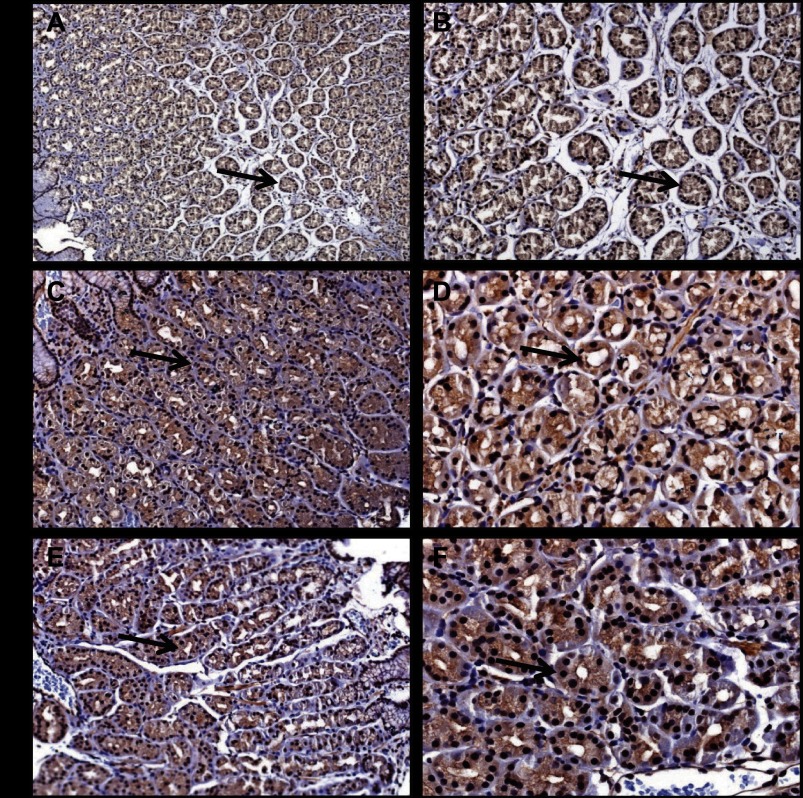
Epithelial localization of PAI-1 in patients on aspirin or NSAIDs. PAI-1 staining (arrow) in epithelial cells in a gastric biopsy from a control subject, *A* ×10, *B* ×20. PAI-1 staining (arrow) in epithelial cells in a gastric biopsy from a subject on low-dose aspirin, *C* ×10, *D* ×20. PAI-1 staining (arrow) in epithelial cells in a gastric biopsy from a subject on NSAIDs, *E* ×10, *F* ×20.

Because PAI-1 may be localized to both epithelial and stromal cells, we then examined the cellular localization of PAI-1 in a subset of patients receiving aspirin or NSAIDs, which included patients both on and off PPIs. In gastric corpus biopsies, there was clear localization of PAI-1 to epithelial cells in gastric glands compatible with expression in parietal cells and chief cells. In contrast, there was only weak staining in the stroma (Fig. 2, *A–F*). The pattern of distribution was similar across patients on aspirin or NSAIDs with or without PPIs and resembled that in control patients, as previously reported (10); the intensity of staining was also similar across groups.

#### PAI-1 expression in epithelial cells is insensitive to indomethacin.

**Fig. 3.**
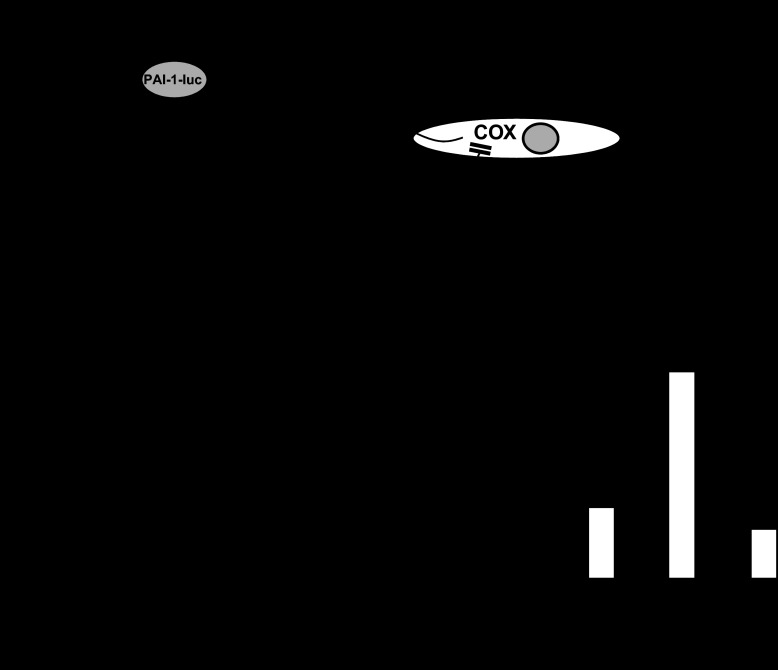
Absence of direct effects of cyclooxygenase (COX) products on PAI-1 expression. *A*: schematic representation of the experimental models used. Epithelial cell lines (AGS-G_R_, MKN45) were transfected with PAI-1 promoter coupled to luciferase (PAI-1-luc) and treated with indomethacin to test the hypothesis that inhibition of COX increased PAI-1-luc (*a*), treated with putative COX products to test the hypothesis that these suppressed PAI-1-luc expression either directly or by inhibiting gastrin-17 (G-17)- or phorbol 12-myristate 13-acetate (PMA)-stimulated responses (also see Table 1) (*b*), and cocultured with myofibroblasts to test the hypothesis that inhibition of COX in these cells stimulated epithelial PAI-1-luc (*c*). PKC, protein kinase C; TXA_2_, thromboxane A_2_. *B*: PAI-1-luc-transfected MKN45 cells exhibit increased expression in response to PMA (100 nM) but no effect of indomethacin (100 mM). *C*: transfected AGS-G_R_ cells exhibit increased luciferase expression in response to G-17 (10 nM) but no effect of indomethacin (100 mM). *D*: PAI-1-luc-transfected AGS-G_R_ responses to G-17, and lack of effect of indomethacin, are not influenced by coculture with gastric myofibroblasts. *P < 0.05, *n* = 6 experiments.

To examine the mechanisms by which aspirin and NSAIDs might regulate PAI-1 expression, we used a reporter consisting of 4.5 kb of the human PAI-1-luc and transfected into MKN45 or AGS-G_R_ cells. We first tested the hypothesis (Fig. 3*A*) that inhibition of COX leads to increased PAI-1 expression. Treatment of MKN45 cells with indomethacin had no effect on PAI-1-luc expression although there was approximately fourfold stimulation of PAI-1-luc by PMA, which was used as a positive control (Fig. 3*B*). Similarly in AGS-G_R_ cells, indomethacin had no effect on PAI-1-luc expression although there was an approximately threefold stimulation by G-17 used as a positive control (Fig. 3*C*). Moreover, indomethacin did not significantly change the response to PMA in MKN45 cells or to G-17 in AGS-G_R_ cells. We then tested an alternative hypothesis (Fig. 3*A*) that COX products were inhibitors of PAI-1 expression. However, the COX products PGE_2_, PGI_2_, and U-46619 (which is a stable ligand of the thromboxane A_2_ receptor) actually had small stimulatory effects on PAI-1-luc expression alone and did not significantly modify the response to PMA (Table 1). Similarly in AGS-G_R_ cells, PGE_2_, PGI_2_, and U-46619 either had no effect or a small stimulatory effect alone and had no effect on the G-17 response with the exception of U-46619, which slightly reduced it (Table 1). The data suggest that the elevated PAI-1 mRNA associated with aspirin or NSAID usage is unlikely to be a consequence of direct suppression of COX in epithelial cells. However, because COX expression also occurs in subepithelial cells, which in general are recognized to regulate epithelial cells by release of a variety of humoral factors, we also considered the possibility that inhibition of COX in these cells might indirectly influence stromal-epithelial cell function to elevate PAI-1. To model this, we added normal human gastric myofibroblasts to AGS-G_R_ cells transfected with PAI-1-luc (Fig. 3*A*). The response of transfected AGS-G_R_ cells to G-17 was unaffected by the presence of myofibroblasts (Fig. *3D*). Moreover, in this model, indomethacin again had no significant effect in influencing PAI-1-luc expression, suggesting that the changes in PAI-1 mRNA seen in vivo do not reflect changes in myofibroblast-epithelial cell signaling.

**Table 1.** Cyclooxygenase products either had no effect or slightly stimulated PAI-1-luc expression in MKN45 and AGS-G_R_ cells

	MKN45 Cells	AGS-G_R_ Cells
Control	1.0 ± 0.13	1.0 ± 0.17
G-17 (10 nM)		3.01 ± 0.12*
PMA (100 ng/ml)	4.14 ± 0.39*	
PGE_2_ (10 μM)	1.82 ± 0.26*	1.73 ± 0.37
PGI_2_ (10 μM)	1.84 ± 0.45*	1.60 ± 0.37
U-46619 (10 μM)	1.42 ± 0.09	1.10 ± 0.31
PGE_2_ + G-17		1.94 ± 0.55*
PGE_2_ + PMA	4.75 ± 0.64*	
PGI_2_ + G-17		2.40 ± 0.56*
PGI_2_ + PMA	3.37 ± 0.28*	
U-46619 + G-17		1.69 ± 0.14^a^
U-46619 + PMA	3.39 ± 0.91*	

Values are means ± SE; *n* = 5 experiments, ANOVA. Stimulation (6 h) of plasminogen activator inhibitor-1 promoter coupled to luciferase (PAI-1-luc)-transfected MKN45 or AGS-G_R_ cells with phorbol 12-myristate, 13-acetate (PMA) or gastrin-17 (G-17), respectively, increased PAI-1-luc expression 3- to 4-fold. PGE_2_, PGI_2_, or a stable ligand of the thromboxane A_2_ receptor U-46619 had either no or a small stimulatory effect on PAI-1-luc expression; in combination with PMA or G-17, as appropriate, PGE_2_, PGI_2_, and U-46619 had no effect or slightly inhibited the response to PMA/G-17.

* *P* < 0.05, statistically significant from control.

a Statistically significant from G-17.

#### Increased PAI-1 mRNA in mouse models of mucosal injury.

**Fig. 4.**
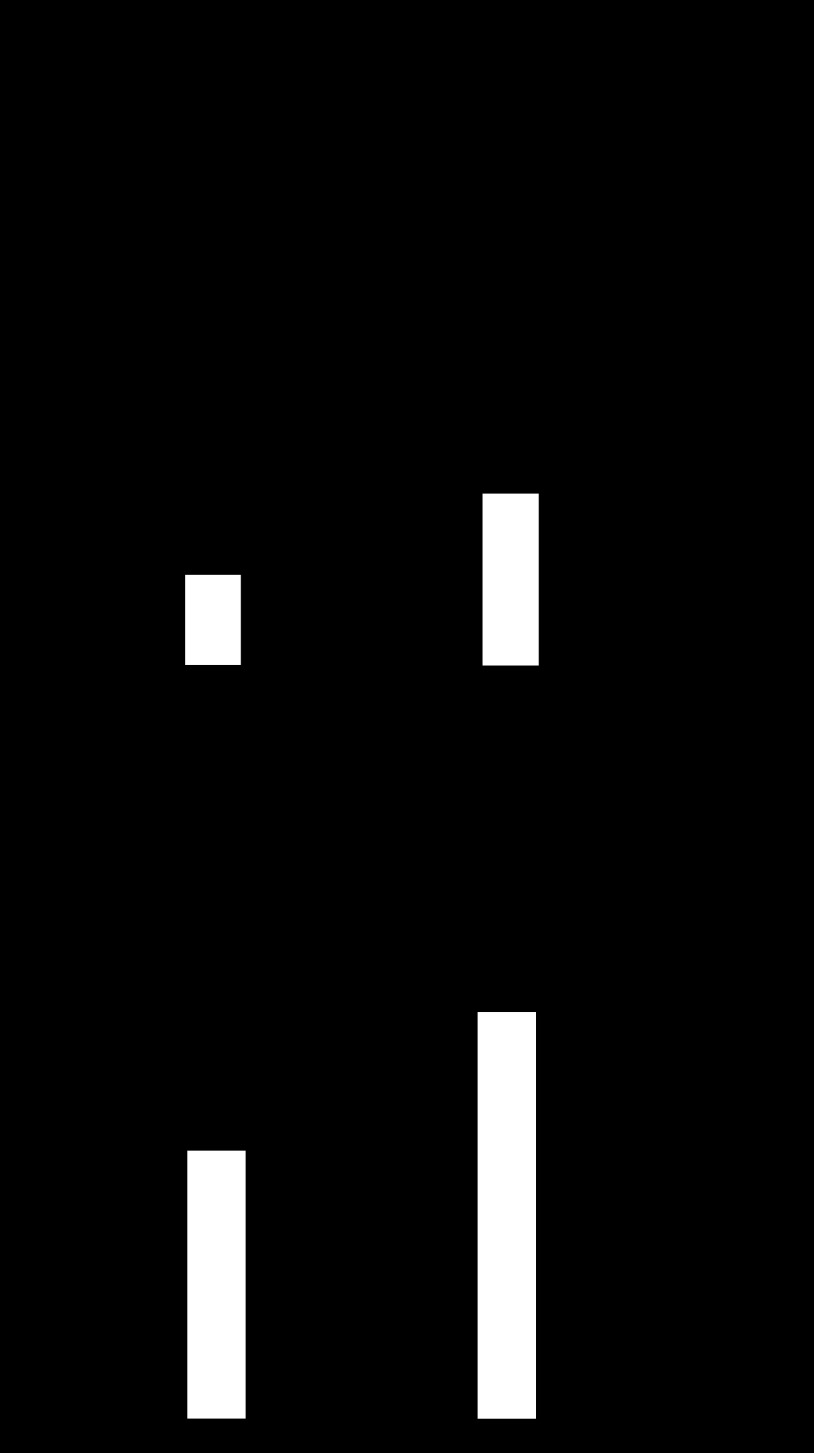
Ethanol and indomethacin increase gastric PAI-1 mRNA abundance in C57BL/6 mice. *A*: increased PAI-1 mRNA relative to GAPDH mRNA in gastric extracts of fasted C57BL/6 mice 1 h after oral administration of absolute ethanol or 6 h after indomethacin compared with vehicle-treated mice. *B*: plasma PAI-1 determined by enzyme-linked immunosorbent assay is similar to control in C57BL/6 mice treated with ethanol 1 h previously or indomethacin 6 h previously. *P < 0.05, *n* = 6, ANOVA.

To determine whether acute experimental mucosal injury in mouse models also regulated PAI-1 expression, we examined mRNA abundance in mouse stomach after either ethanol or indomethacin at similar time points to those often used for assessment of mucosal damage. Thus 1 h after absolute ethanol, there was a 4.5-fold increase in PAI-1 mRNA, and 6 h after indomethacin there was a 2-fold increase in relative mRNA abundance (Fig. 4*A*). Plasma PAI-1 concentrations measured by ELISA were not significantly different in ethanol- or indomethacin-treated mice compared with the relevant vehicles (Fig. 4*B*), although in both cases there were increased hemorrhagic lesions in the treated animals (see below).

#### Gastroprotective effects of PAI-1.

**Fig. 5.**
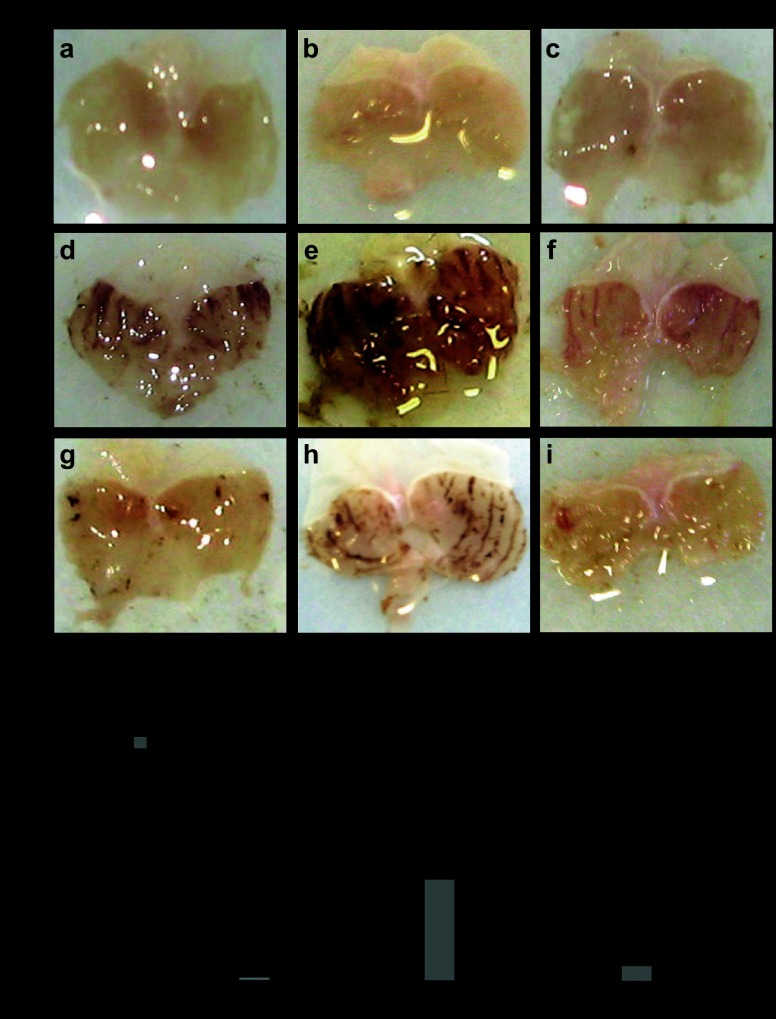
Protective effect of PAI-1 against ethanol-induced gastric lesions. *A*: representative photographs of gastric hemorrhagic lesions formed in C57BL/6, PAI-1^−/−^, and PAI-1-H/Kβ mice 1 h after gavage of 100 μl saline (*a–c*), absolute ethanol (*d–f*), or 50% ethanol (*g–i*). *B*: quantification of gastric hemorrhagic lesions in C57BL/6, PAI-1^−/−^, and PAI-1-H/Kβ mice 1 h after treatment with saline, absolute ethanol, or 50% ethanol. *P < 0.05, *n* = 12–22, ANOVA.

**Fig. 6.**
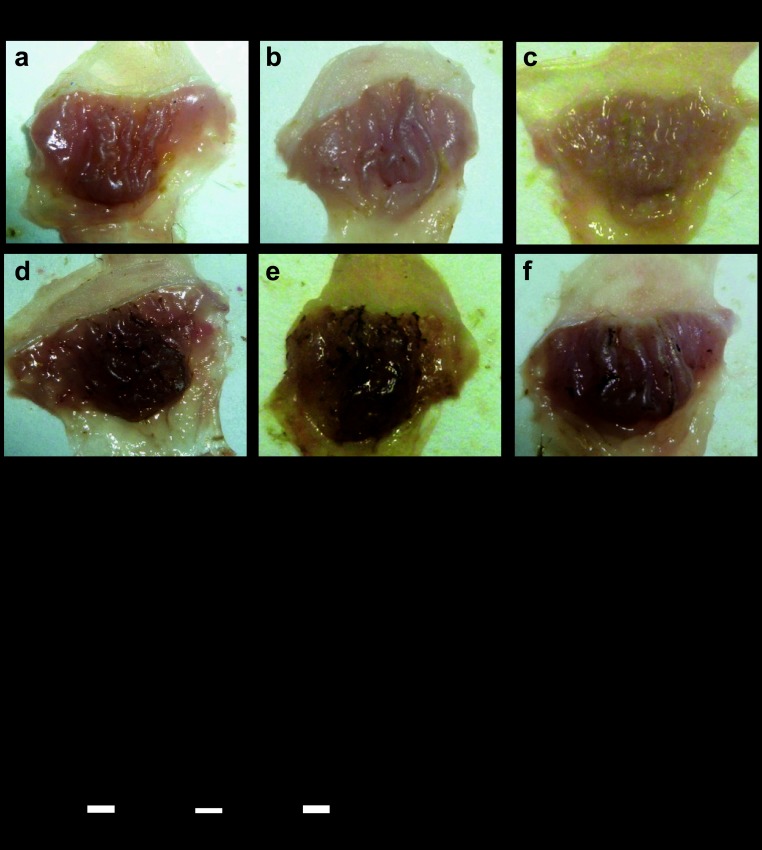
Protective effect of PAI-1 against indomethacin-induced gastric lesions. *A*: representative photographs of gastric hemorrhagic lesions formed in C57BL/6, PAI-1^−/−^, and PAI-1-H/Kβ mice 6 h after treatment with vehicle (*a–c*) or 20 mg/kg indomethacin (*d–f*) by gavage. *B*: quantification of gastric hemorrhagic lesions formed in C57BL/6, PAI-1^−/−^, and PAI-1-H/Kβ mice after indomethacin. *P < 0.05, *n* = 8–27, ANOVA.

Finally, we asked whether expression of gastric PAI-1 influenced lesion development in models of mucosal injury using mouse strains that are either null for PAI-1 or in which PAI-1 is overexpressed in gastric parietal cells (11). Interestingly, in mice null for PAI-1, we demonstrated increased hemorrhagic lesions in response to both absolute ethanol and 50% ethanol compared with C57BL/6 mice. In contrast, in PAI-1-H/Kβ mice in which there is targeted expression of PAI-1 to gastric parietal cells, lesions were reduced in response to absolute ethanol (Fig. 5, *A* and *B*). Second, we examined gastric lesions in response to indomethacin. Again, the lesions in PAI-1 null mice were greater than those in wild-type mice, whereas those in PAI-1-H/Kβ mice were reduced compared with wild-type mice (Fig. 6, *A* and *B*). There was no difference between male and female mice in either model (results not shown), and the data for both sexes were therefore pooled. The findings suggest that gastric expression of PAI-1 protects against acute mucosal damage.

## DISCUSSION

The main findings of this study are that aspirin or NSAID usage is associated with increased gastric mucosal PAI-1, but not uPA or PAI-2, mRNA and that expression of PAI-1 in the stomach protects against ethanol- and indomethacin-induced hemorrhagic lesions. Together these observations suggest that PAI-1 expression is part of a defense response to mucosal damage that follows aspirin or NSAID exposure. The gastric mucosal protective effects of PAI-1 are seen in two different animal models of early (1–6 h) responses to damage. The data are compatible with actions of PAI-1 to stabilize fibrin clots by inhibition of uPA or tPA and so promote hemostasis and minimize the formation of hemorrhagic lesions.

In examining PAI-1 expression in response to aspirin and NSAID usage, we adopted a cross-sectional approach. Patients taking aspirin were all on a low dose (75 mg/day), but the NSAID group was relatively heterogeneous (44% were on ibuprofen and 25% on diclofenac). There was also variation in PPIs consumed (many were on lansoprazole or omeprazole), but, although this might increase gastric PAI-1 mRNA in normal subjects, it appears not to further increase PAI-1 mRNA in subjects on aspirin or NSAIDs. We considered the present approach justified given *1*) that there appear to have been no previous studies of aspirin or NSAID effects on PAI-1 in the human stomach, and *2*) prior to this study, the scientific and ethical justification for a prospective randomized trial using defined doses of aspirin or NSAIDs in patients presenting for gastroscopy would have been weak. In the case of NSAIDs in particular, it would now be useful to conduct such a trial not least to clarify whether there is indeed significantly increased gastric PAI-1 in patients on NSAIDs plus PPIs.

The changes observed in PAI-1 are selective in that there were not comparable changes in uPA or PAI-2 mRNA. Moreover, as with previous studies of PAI-1 in *H. pylori* infection or hypergastrinemic subjects (10, 18), the expression was predominantly localized to major gland cell types. Although not specifically explored in this study, PAI-1 expression has previously been found in chief, parietal, and enterochromaffin-like cells (10), and the pattern of distribution observed here, i.e., in most glandular cells, is compatible with this.

The present analysis was based on *H. pylori*-negative patients with normal gastric histology. There is now obviously scope for future studies to examine PAI-1 expression in, for example, patients with gastric ulcer. Previous studies have shown increased PAI-1 in peptic ulcer (25), and, in a group of 35 gastric ulcer patients, we have also found elevated PAI-1 mRNA in gastric corpus biopsies (unpublished observations). However, the interpretation of these observations is complicated by the fact that a significant proportion of gastric ulcer patients have elevated circulating gastrin concentrations (particularly G-34) (20), and most are *H. pylori* positive. Thus, appropriately powered studies of gastric ulcer that take into account the known variables influencing gastric PAI-1 expression are now required.

There is widespread expression of PAI-1 in many different tissues, including liver, adipose stromal tissue, platelets, and endothelial cells, and increased expression is a feature of inflammation and tissue damage. There are likely to be multiple mediators of increased PAI-1 expression of which transforming growth factor-β is a well-studied example. In the intestine, there is increased PAI-1 in radiation damage (1), experimental colitis (7), and in neurons in Crohn's disease (12). In the case of the stomach, there is evidence of increased expression in gastric cancer that is associated with poor outcome (2, 17); moreover, there are direct effects of gastrin, and of *H. pylori*, in inducing PAI-1 in epithelial cells (9, 10, 18). In contrast, the present data indicated that indomethacin did not directly influence PAI-1 expression in epithelial cell lines and that COX products such as PGE_2_, PGI_2_, and thromboxane are unlikely to have robust inhibitory actions on expression, as might be predicted by the in vivo response to aspirin or NSAIDs. One important difference between in vivo and in vitro analyses is that subepithelial cells are excluded in the latter. Previously, PGE_2_ was shown to stimulate uPA and uPA receptor expression in gastric fibroblasts, and indomethacin inhibited expression (8). We attempted to model possible epithelial/subepithelial cell interactions by including myofibroblasts together with PAI-1-luc-transfected epithelial cells, since myofibroblasts produce growth factors regulating epithelial cell function, are implicated in wound healing, and may also express COX. However, the results did not suggest a myofibroblast component in the PAI-1 response to NSAIDs. Clearly, it remains possible that other stromal cells, for example, inflammatory or immune cells, are required for epithelial PAI-1 responses to NSAIDs; in the future, it would be useful to test this idea preferably using primary human gastric epithelial cells. Nevertheless, the present data support the idea that increased gastric PAI-1 mRNA abundance in vivo is either a consequence of indirect tissue responses to COX inhibition by aspirin or NSAIDs, or of other effects of these treatments that are not COX-mediated.

The biological actions exerted by PAI-1 are complex, not least because they involve both uPA- and tPA-dependent and -independent effects exerted over differing time scales. In the present animal models, PAI-1 is able to influence the formation of hemorrhagic lesions over periods of just a few hours. One plausible explanation for the protective effect of PAI-1 in these circumstances is that it helps stabilize fibrin clots and promote hemostasis. Importantly, there was no significant difference in plasma PAI-1 concentration in mice after acute administration of ethanol or indomethacin even though gastric mRNA concentrations were increased; the protective effect of PAI-1 in the stomach would appear therefore to reflect a local rather than systemic effect.

It is known that PAI-1 inhibits epithelial cell migration and proliferation, but it seems unlikely that these targets would account for the present findings (10, 18). Moreover, the present observations on mucosal protection are clearly distinct from longer-term effects of PAI-1, for example, in promoting fibrogenesis as occurs in other organs (14). Interestingly, another adipokine, adiponectin, has also been reported recently to have a protective effect against ethanol ulcer, suggesting links between adipokines and gastric mucosal function that are wider than previously supposed (26).

A gastrointestinal phenotype in PAI-1 null mice has not previously been described although we have recently shown that, in response to *H. felis* infection, gastric mucosal thickness and inflammation is similar to that in C57BL/6 mice (11). The impaired mucosal protection in PAI-1 null mice contrasted with the enhanced protection in mice that selectively overexpress PAI-1 in the stomach. The latter were produced by targeting transgenic PAI-1 to parietal cells using the promoter of the H^+^-K^+^-ATPase β-subunit, which is selectively expressed in these cells (14). Expression of the transgene leads to approximately threefold higher total PAI-1 mRNA (i.e., wild type and transgene together) in gastric mucosa. Because the increase in PAI-1 mRNA in response to aspirin, NSAIDs, or ethanol in humans and mice is two- to sixfold higher than control, it would seem that the PAI-1-H/Kβ mice have elevated expression over a functionally relevant dynamic range. The PAI-1-H/Kβ mice exhibit moderate hyperphagia and obesity probably because of resistance to the satiating actions of cholecystokinin (CCK) by suppression of vagal afferent stimulation (11). There is evidence for CCK-vagal interactions mediating gastroprotective responses to ethanol but not aspirin-induced lesions (3). Because PAI-1 null mice were susceptible to both ethanol and indomethacin, and PAI-1-H/Kβ mice were protected in both cases, it seems unlikely that these effects are secondary to modulation of CCK-vagal pathways.

It has been clear for many years that plasmin plays a role in ulcer bleeding. Wodzinski et al. showed increased uPA and PAI-1 at the edge of gastric and duodenal peptic ulcers (25), and then Vreeburg et al. showed that there is increased fibrinolytic activity in patients with bleeding peptic ulcer, which is decreased by acid suppression at least in part by increased plasma PAI-1 (23). Taken in conjunction with the present results, it appears therefore that PAI-1 acts to restrain fibrinolysis as part of an autoregulatory loop activated by mucosal injury. A strategy to selectively increase gastric epithelial PAI-1 might therefore be therapeutically useful in preventing ulcerogenesis in the relevant patients.

## GRANTS

This work was supported by grants from the National Institute for Health Research (NIHR)-funded Liverpool Biomedical Research Centre for Microbial Disease and the Wellcome Trust. S. Lyons was supported by a Biotechnology and Biological Sciences Research Council research studentship; A. R. Moore was a NIHR fellow.

## DISCLOSURES

No conflicts of interest, financial or otherwise, are declared by the authors.

## AUTHOR CONTRIBUTIONS

Author contributions: S.K., I.S., S.L., R.D., D.M.P., A.V., and G.J.D. conception and design of research; S.K., I.S., S.L., A.R.M., S.V.M., L.T., and R.D. performed experiments; S.K., I.S., S.L., A.R.M., S.V.M., L.T., R.D., D.M.P., A.V., and G.J.D. analyzed data; S.K., I.S., S.L., A.R.M., S.V.M., L.T., R.D., D.M.P., A.V., and G.J.D. interpreted results of experiments; S.K. prepared figures; S.K., I.S., R.D., D.M.P., A.V., and G.J.D. edited and revised manuscript; S.K., I.S., S.L., A.R.M., S.V.M., L.T., R.D., D.M.P., A.V., and G.J.D. approved final version of manuscript; G.J.D. drafted manuscript.
